# Image-Guided Proton Therapy for Elderly Patients with Hepatocellular Carcinoma: High Local Control and Quality of Life Preservation

**DOI:** 10.3390/cancers13020219

**Published:** 2021-01-09

**Authors:** Hiromitsu Iwata, Hiroyuki Ogino, Yukiko Hattori, Koichiro Nakajima, Kento Nomura, Kensuke Hayashi, Toshiyuki Toshito, Shigeru Sasaki, Shingo Hashimoto, Jun-etsu Mizoe, Yuta Shibamoto

**Affiliations:** 1 Department of Radiation Oncology, Nagoya Proton Therapy Center, Nagoya City West Medical Center, Nagoya 462-8508, Japan; h-iwa-ncu@nifty.com (H.I.); yhattori7@yahoo.co.jp (Y.H.); koichiro1288@hotmail.co.jp (K.N.); knomura01i04m08t@gmail.com (K.N.); 2 Department of Radiology, Nagoya City University Graduate School of Medical Sciences, Nagoya 467-8601, Japan; hashimoto.ncu@gmail.com (S.H.); yshiba@med.nagoya-cu.ac.jp (Y.S.); 3 Department of Proton Therapy Technology, Nagoya Proton Therapy Center, Nagoya 462-8508, Japan; k.hayashi.12@west-med.jp; 4 Department of Proton Therapy Physics, Nagoya Proton Therapy Center, Nagoya 462-8508, Japan; t.toshitou.20@west-med.jp; 5 Department of Diagnostic Radiology, Nagoya City West Medical Center, Nagoya 462-8508, Japan; ssasaki916@yahoo.co.jp; 6 Sapporo High Functioning Radiotherapy Center, Hokkaido Ohno Memorial Hospital, Sapporo 063-0052, Japan; junetsumizoe@gmail.com

**Keywords:** image-guided proton therapy, hepatocellular carcinoma, overall survival, quality of life, elderly patients

## Abstract

**Simple Summary:**

The number of very elderly patients with hepatocellular carcinoma (HCC) aged 80 years and older has been steadily increasing with extensions in life expectancy due to improvements in medication and healthcare, and many recent studies focused on this specific population. Since these patients often have a higher incidence of associated co-morbidities, and improving or maintaining their quality of life is important, minimally invasive treatment is warranted. We retrospectively investigated the efficacy and safety of image-guided proton therapy (IGPT) for elderly HCC patients. IGPT was safe and considered effective for HCC in elderly patients. Since there was no worsening of quality of life during and after treatment, IGPT may cure HCC with a high probability without changing the daily living of elderly patients.

**Abstract:**

This study retrospectively investigated the efficacy and safety of image-guided proton therapy (IGPT) for elderly (≥80 years old) hepatocellular carcinoma (HCC) patients. Proton therapy was performed using respiratory-gated and image-guided techniques. Seventy-one elderly HCC patients were treated using IGPT. The Child–Pugh score was A5 in 49 patients, A6 in 15, and B7-9 in 7. Forty-seven patients with a peripherally located tumor were administered 66 gray relative biological effectiveness (GyRBE) in 10 fractions, whereas 24 with a centrally located tumor received 72.6 GyRBE in 22 fractions. The median follow-up period of surviving patients was 33 months (range: 9–68). Two-year overall survival (OS) and local control (LC) rates estimated by the Kaplan–Meier method were 76% (95% confidence interval: 66–87%) and 88% (80–97%), respectively. According to the Common Terminology Criteria for Adverse Events version 4.0, no grade 2 or higher radiation-induced liver disease was observed, and only 1 patient developed grade 3 dermatitis. The quality of life score (European Organization for Research and Treatment of Cancer (EORTC) QLQ-C30 version 3.0, QLQ-HCC18, and SF-36) did not change after 1 year, except for the three-mental component summary (SF-36, improvement). IGPT is a safe and effective treatment for HCC in elderly patients.

## 1. Introduction

The number of very elderly patients with hepatocellular carcinoma (HCC) aged 80 years and older has been steadily increasing with extensions in life expectancy due to improvements in medication and healthcare [1], and many recent studies focused on this specific population [2]. The new definition of elderly includes individuals aged 80 years or older [3], who often have a higher incidence of associated co-morbidities. The management of malignant diseases in elderly patients has become a global issue in the aging society [4]. Japan has the most aged society worldwide. An increased proportion of elderly HCC patients has been reported due to the prevalence of non-alcoholic fatty liver disease (NAFLD), particularly non-alcoholic steatohepatitis (NASH), caused by changes in dietary habits worldwide. In addition to these etiological factors, the rapid aging of hepatitis C patients has contributed to the increase in elderly HCC according to a Japanese nationwide survey [5].

Hepatic resection (HR) is a first-choice treatment for HCC. Previous studies reported its safety and usefulness [5,6,7], whereas others found a higher incidence of complications and mortality rates in elderly patients [8,9]. Therefore, a reduction in the level of invasiveness needs to be considered by selecting a more defensive surgical technique, in addition to carefully assessing heart/lung functions, other concomitant malignant tumors, and preoperative liver function [5,6,10]. According to the above-mentioned Japanese nationwide survey, surgery was only performed on approximately 31% of HCC patients; therefore, minimally invasive treatment is considered more appropriate for the remaining patients. However, among the factors used to define optimal treatment for HCC, age is not included in the guidelines. This current situation highlights the need to investigate optimal treatments for elderly HCC. Alternative therapies to surgery include radiofrequency ablation (RFA), microwave ablation (MWA), and transcatheter arterial chemoembolization (TACE). While many studies reported the usefulness of these therapies, the Liver Cancer Study Group of Japan (LCSGJ) found lower survival rates after these alternative therapies than after HR [5]. However, this study did not investigate adverse events and inoperable patients were treated by RFA. Therefore, there may have been differences in co-morbidities and liver function. Furthermore, it is important to note that RFA cannot necessarily be performed in all patients due to invisibility on ultrasonography.

The treatment of elderly patients is also important for improving or maintaining quality of life (QOL) [11]. Since the recurrence rate within 2 years of the diagnosis of HCC is high, relapse and treatment are often repeated [12], which may deteriorate QOL. We hypothesized that health-related QOL (HRQOL) may decrease in elderly patients due to complications and muscular weakness. The QOL scores of the European Organization for Research and Treatment of Cancer (EORTC) QLQ-C30 version 3.0 and QLQ-HCC18 were identified as prognostic factors for overall survival (OS) [13,14]; however, few studies included elderly patients.

As an alternative to HR and RFA, recent studies reported favorable outcomes for small HCC treated by stereotactic body radiotherapy (SBRT) [15,16]. However, few studies have reported SBRT for HCC in elderly patients, and LCSGJ did not compare SBRT with other procedures. Furthermore, few studies have investigated changes in QOL [5]. The efficacy of proton therapy (PT) for HCC has been demonstrated [17,18]. The surrounding liver radiation dose is lower in PT than in SBRT, and, thus, hepatotoxicity is low. The preservation of remnant liver function is indispensable for the success of a second treatment for new lesions developing due to hepatitis and cirrhosis, multicentric occurrence, and intrahepatic metastasis. Although PT provides a more focused dose distribution to the tumor than SBRT, its routine use in the treatment of elderly HCC remains controversial because they often have many complications and extensive treatment history. The aim of the present study was to evaluate the efficacy and safety of image-guided PT (IGPT) for elderly HCC patients.

## 2. Results

### 2.1. Patient and Treatment Characteristics

Of 473 HCC patients treated using IGPT, 71 met the inclusion criteria. Patient characteristics are summarized in Table 1. Fifteen BCLC stage C patients with PS 1-2 and 3 stage D patients with PS 3 were included, because our cancer board recommended to treat their bulky tumors to avoid cancer death due to tumor rupture and massive bleeding. Regarding liver function, more than half of the patients had subnormal values. Treatment characteristics and dose volume analyses are summarized in Table 2. Forty-seven patients with a peripherally located tumor were administered 66 gray (Gy) relative biological effectiveness (RBE) in 10 fractions and 24 with a central tumor received 72.6 gray relative biological effectiveness (GyRBE) in 22 fractions. A liver volume of 35% or more of the standard liver volume was spared (unirradiated) while satisfying PTV coverage in all but six patients; these six patients met the liver constraints, but did not satisfy the PTV coverage criteria due to their tumor being adjacent to the intestinal tract and or having a large volume.

### 2.2. Disease Control and Survival

**Figure 1:**
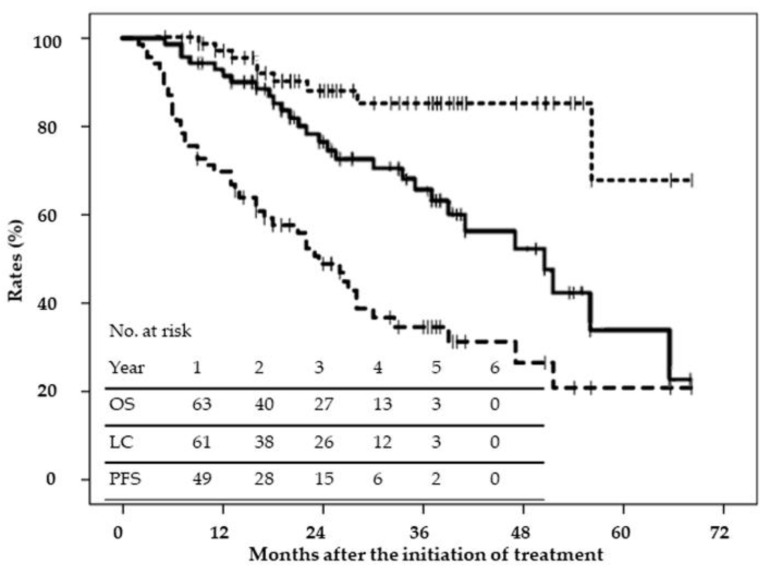
Curves for overall survival (OS) (solid line), local control (LC) (dotted line), and progression-free survival (PFS) (dashed line) for all patients.

**Figure 2:**
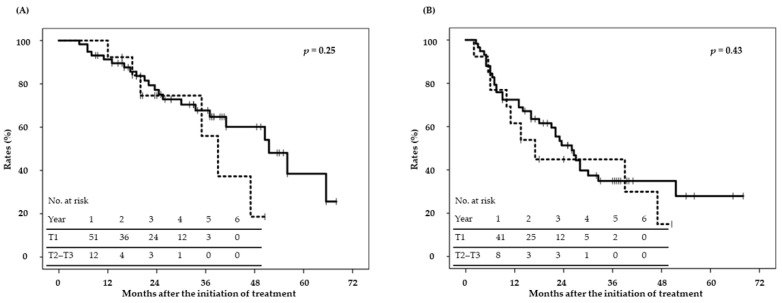
(**A**) OS and (**B**) PFS according to TNM stage. Solid line, T1; dotted line, T2–T3.

**Figure 3:**
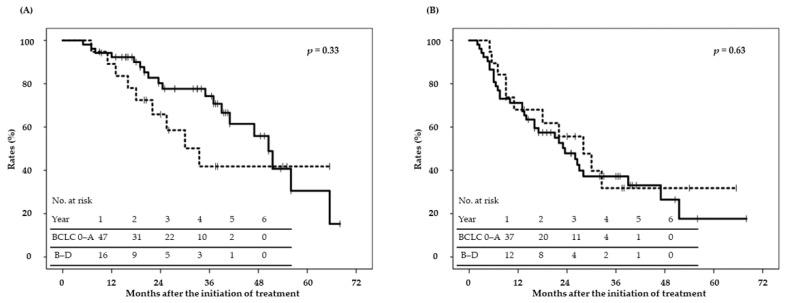
(**A**) OS and (**B**) PFS according to BCLC stage. Solid line, BCLC 0–A; dotted line, B–D.

The median follow-up period of surviving patients was 33 months (range: 9–68 months). OS, local control (LC), and progression-free survival (PFS) rates are shown in Figure 1. Two-year OS, LC, and PFS rates were 76% (95% CI; 66–87%), 88% (80–97%), and 50% (38–62%), respectively. Median LC was not reached, and median OS and PFS were 49 and 24 months, respectively. OS differed according to sex, performance status, treatment history, the Child–Pugh classification, operability, alpha fetoprotein levels, and protein induced by vitamin K absence or antagonist-II (PIVKA-II) levels in the univariate analysis (Table 3); however, only sex and the performance status were associated in the multivariate analysis (Table 3). Figure 2 shows OS and PFS curves according to the TNM stage (T1 vs. T2–T3); there were no significant differences in OS and PFS between T1 and T2–T3. Figure 3 shows OS and PFS curves according to the BCLC stage (0–A vs. B–D); there were no significant differences in OS and PFS between BCLC 0-A and B-D. Local recurrence was detected in nine patients with large tumors close to the gastrointestinal tract; recurrence may have been due, in part, to the radiation dose being reduced to a tolerable level. Twenty-six patients developed intrahepatic metastasis or multicentric occurrence. In addition, 11 patients developed lymph node, bone, or lung metastasis or peritoneal dissemination. Twenty-eight patients died, 15 of whom died of other diseases. LC differed with the tumor diameter, tumor volume, and vicinity to the gastrointestinal tract in the univariate analysis; however, none of the factors were associated with LC in the multivariate analysis (Table 4).

### 2.3. Complications and QOL Scores

Major complications are summarized in Table 5. No grade 2 or higher radiation-induced liver disease was observed, and only one case of grade 3 dermatitis was noted. No worsening of the ALBI grade from 1 or 2 to 3 was found within 3 months after IGPT. Except for disease progression cases, no significant decrease was observed in the Child–Pugh score of ≥2. Changes in EORTC QLQ-C30, QLQ-HCC-18, and SF-36 1 year after treatment are shown in Table 6. Emotional and social functioning scores improved at 1 year. All QOL scores were favorable. No significant differences were observed in QOL scores after 1 year, except in the three-mental component summary (3-MCS, SF-36, improvement). There were no significant changes in QOL scores during the 1-year follow-up in the one-way analysis of variance. The SF-36 subscale score was ≥50; QOL was higher than in the Japanese population or scoring with respect to age. The three-physical component summary (3-PCS), three-role-social component summary (3-RCS), and two-physical component summary (2-PCS) scores were ≤50, which were lower than the national standard value before treatment. However, there was no significant decrease after treatment. Fever transiently deteriorated at 6 months for unknown reasons. In 19 BCLC stage B-D patients, no significant changes were also observed in QOL scores after 1 year.

## 3. Discussion

To the best of our knowledge, this is the first study on the feasibility and effectiveness of IGPT for elderly HCC in Japan. At diagnosis, surgery was possible in approximately 40% of patients and RFA in approximately 20%; therefore, the indication of radical local therapy was limited. Regarding liver function, the Child–Pugh score was A5 in approximately 70% of patients, whereas normal liver function was not maintained in ≥50% based on ICGR_15_, the ALBI grade, ^99m^Tc-GSA hepatic scintigraphy findings, and the FIB-4 index. As age-related changes in liver function occurred, the coagulation factor level was low, the synthesis of proteins, such as amino acids and albumin, was reduced, and a decrease in bile secretion and delayed bile excretion were observed; however, the levels of transaminase and alkaline phosphatase did not appear to be affected by aging [19,20]. Decreases in liver weight/volume related to a reduction in the hepatic blood flow volume were previously reported as morphological changes in the liver [21]. In addition, ICG test abnormalities and a reduction in portal blood flow were noted. Therefore, whether the posttreatment hepatic reserve may be maintained under poor basic conditions needs to be considered when selecting a suitable treatment among limited options. Many elderly patients previously received treatment. In this study, approximately 35% patients had received prior treatment. In addition, treatment is continued from secondary to tertiary treatment in many cases. Therefore, the residual liver volume is important, and IGPT is more advantageous than SBRT [22]. Although it currently remains unclear whether low-dose irradiation to the normal liver is acceptable, the concept of the standard liver volume was also adopted in this treatment plan, as in the case of surgery. In a previous study on the causes of radiation-induced liver disease (RILD), the standard liver volume was more important than the mean liver dose or V20 [23]. In the present study, no grade 2 or higher classical or nonclassical RILD developed despite the lower liver function. This may have been because the standard liver volume was included in planning constraints (Liver-GTV volume receiving ≤ 1 GyRBE exceeds 35% of the standard liver volume) to protect the residual liver, and may also have accounted for the differences observed in the incidence of RILD between IGPT and SBRT. In addition, although 25 patients had a tumor in proximity (< 1 cm) to the gastrointestinal tract, no grade 2–3 gastrointestinal toxicities were observed. As shown in Table 2, the target dose near the gastrointestinal tract was reduced, and so the dose constraint on the gastrointestinal tract for this study was considered safe and reasonable (Table S1).

OS in the present study was considered favorable when compared to OS after other treatment modalities. Uni- and multivariate analyses revealed that PS and previous treatment were significant factors, whereas a difference in liver function was not. The number of patients was <100 and, thus, a larger number of patients needs to be investigated in the future. Representative outcomes of surgery, SBRT, and other modalities for elderly HCC report that 5-year OS ranged between 29 and 60% and grade 3 or higher morbidities between 3 and 30%, while post-operative mortalities ranged between 0 and 5% [5,6,7,24,25,26,27,28]. Previous findings on HR in elderly patients vary. Although several studies reported favorable outcomes, there may have been a bias; patients with better liver function, fewer complications, and no other malignant tumors, may have been selected for the surgery group. Furthermore, less invasive surgery than HR may have been performed [5]. The risk of surgery-associated death is high (approximately 5%). Post-operative complications associated with blood loss, the duration of surgery, and decreasing albumin levels were reported to affect the prognosis of elderly patients [6,29]. In addition, surgery-associated complications have been suggested to cause protracted inflammation or immune hypofunction, reducing the survival rate and resulting in the enlargement of HCC or malignant tumors in other organs/micrometastasis [8]. Postoperative delirium has recently been emphasized. Its incidence in elderly patients with HCC is reportedly about 75%, which is nearly four-fold higher than in young patients [7,30]. Typical postoperative delirium rapidly induces psychomotor excitation and hallucination/delusion during the hours in which consciousness is clear 2 to 5 days after surgery, often causing problematic behaviors, such as the self-removal of a catheter or tube for intravenous drips, difficulties in resting maintenance, and day and night reversal. Perioperative delirium is associated with a higher risk of postoperative complications, which increases the mortality rate [30,31]. IGPT does not require long-term admission and, thus, these risks may be avoided.

QOL evaluations and cost effectiveness are important issues when comparing treatment modalities for elderly HCC. Controversy surrounds the use of costly PT for elderly patients [32]. In the present study, as a pretreatment HRQOL score, physical function was low in the QLQC-30 and HCC18 evaluation, and the scores of symptom items, including fatigue, were high; some symptoms were present. SF-36 scores can be compared with the national standard values [33]. Based on SF-36, PCS and RCS scores were markedly low; physical and social aspects were both poorer than those in healthy adults. The values for subscales, such as physical aspects, were slightly better than age-related scores in individuals aged 80 to 84 years; however, scores for general health and vitality were slightly lower, and PCS, MCS, and RCS scores were similar [33]. Patients receiving PT generally have a high level of motivation to undergo treatment with a relatively high income; this may cause bias. However, based on SF-36 data, scores in patients were similar to those in healthy adults of the same age. SF-36 subscale scores were lower than those for HCC patients who underwent surgery, suggesting lower PCS and RCS scores [34]. The admission period for surgery is approximately 3 weeks and physical strength may decrease after discharge. In consideration of liver protection, high-intensity physical activities are avoided and body-caring recuperation behaviors may reduce physical strength after surgery. Furthermore, daily living may be hindered due to physical or mental reasons: admission, the avoidance of opportunities to drink alcohol, and mental stress.

Recovery was achieved 6 to 12 months after surgery at an operable age. However, no study reported changes in QOL data in elderly patients. The present results revealed no reduction in physical function/social aspects or symptom appearance 6 to 12 months after IGPT. As a rule, outpatient care is possible and the incidence of adverse events after treatment is low. In addition, rehabilitation due to muscle weakness is not required, in contrast to HR [35]; therefore, there may be no reduction in HRQOL early after irradiation. Early data were not obtained in the present study, and this is a limitation. In elderly patients, muscular exhaustion related to aging or inflammatory disease, a hepatopathy-related reduction in protein synthesis, and a decrease in liver glycogen storage may induce skeletal muscle collapse [36]. Muscles are considered to be the second liver, and muscle-volume maintenance is important for ammonia detoxification or energy source synthesis in elderly patients with HCC. Therefore, IGPT may also be more advantageous than surgery from the viewpoint of aspects early after treatment. In addition, 10 and 30% of patients were classified as Child–Pugh B and Liver damage B or C, respectively, whereas reduced QOL, as reported in SBRT [37], was generally not observed. This may be partially explained by low liver toxicity. Surgery improved environmental QOL, but reduced physical domain scores [38], whereas RFA more significantly ameliorated dyspnea, appetite loss, and negative body image than embolization and surgery [39]. IGPT may be conducted without influencing a patient’s life and work during and after the treatment period. Expenditures for hospital care, the treatment of adverse reactions and complications, and rehabilitation cannot be directly estimated, but may be acceptable for elderly patients. However, there are race-related differences in QOL, and the quality-adjusted life year, involving cost-effectiveness, needs to be compared with that for other modalities.

## 4. Materials and Methods

### 4.1. Study Design and Patient Eligibility

This retrospective study included elderly HCC patients treated using IGPT between June 2013 and December 2019, and was approved by the Institutional Review Board. The registry number was 12-02-24 (20). Study endpoints were the OS rate, LC rate, PFS rate, incidence of adverse events, and changes in QOL scores. Inclusion criteria were: (1) histologically confirmed or image-diagnosed primary HCC staged as T1, T2, or T3N0M0 (UICC TNM 8th ed.)-the latter patients had to have typical findings, including early enhancement and wash out at a late phase on CT and a hepatobiliary phase on magnetic resonance imaging (MRI); (2) one or two liver tumors; (3) age, 80 years or older; (4) no previous radiotherapy around the lesion; (5) ECOG-PS ≤ 3; (6) Child–Pugh classification A5–B9; (7) dose constraints of the organs at risk achievable within the tolerable dose; (8) follow-up ≥6 months or until death; and (9) written informed consent. Possibility of hepatic resection (partial resection, segmental resection, lobectomy, etc.) was discussed on the in-hospital cancer board based on liver function, residual liver volume, tumor site/size, and patient conditions. Possibility of RFA was judged on the cancer board based on the position and size of the tumor, distance to the surrounding blood vessels, visibility in ultrasonography, and patient conditions. Possibility of TACE was also judged on the board based on the renal function, status of hepatofugal portal blood flow, history of recent bleeding from esophageal varices, and status of portal-systemic shunt. Indications for surgery, RFA, and TACE were determined based on these criteria and were instructed to the patients.

### 4.2. Treatment Protocols and Systems

Tumors for which the common bile duct was included in the irradiation field were defined as centrally located tumors and other tumors were defined as peripherally located tumors. For peripherally located and centrally located tumors, the prescribed dose to the isocenter was 66 GyRBE in 10 fractions and 72.6 GyRBE in 22 fractions, respectively. All irradiation was performed once a day, 5 days a week, using 2-4 beam portals. We adopted the RBE value of 1.1 [40]. Our treatment machines and systems (PROBEAT III (Hitachi, Ltd., Tokyo, Japan) and VQA (Hitachi, Ltd., Tokyo, Japan)) were previously described in detail [41,42]. Since scanning irradiation for elderly HCC could increase uncertainty due to inadequate breath-holding and disturbed respiratory waveforms, a passive scattering technique using a range modulation wheel with mainly 120- to 200-MeV proton beams was employed for all treatments. The image-guided technique was previously described in detail [42]. Briefly, a transarterial or percutaneous fiducial marker (5-mm-long, 0.018-inch-diameter straight microcoils: Hilal Embolization Microcoil ^TM^, Cook, Bloomington, IN, USA or Gold Anchor ^TM^, Naslund Medical AB, Huddinge, Sweden) was implanted near the tumor prior to treatment [43]. Daily patient alignments were performed with 2D/2D matching methods using the PIAS system (Hitachi, Ltd., Tokyo, Japan), and were achieved by matching fiducial markers and/or vertebral bones. The translation and rotation of the patient were compensated for by adjustments to the treatment couch. For the set-up, the fiducial markers on the digitally reconstructed radiographs had to agree within 2 mm.

### 4.3. Treatment Planning

Our method was previously described in detail [18,42]. The details of treatment planning are shown in File S1 [44]. Dose constraints for normal tissues were set based on data obtained from previous analyses [45] and created in consideration of liver function. In particular, the Liver-GTV volume receiving ≤1 GyRBE was set to exceed 35% of the standard liver volume (706.2 × body surface area (m^2^) + 2.4 (mL)) [46] (Table S1) [45,47]. The severity of cirrhosis was assessed by referencing the Child–Pugh classification, ICGR15 (indocyanine green retention rate at 15 min), and ^99m^Tc-GSA hepatic scintigraphy. The treatment plan that covered PTV with 95% or more of the prescribed dose was desirable. However, the goal was to achieve treatment encompassing 95% of ICTV with 98% or more of the prescribed dose because many tumors were located adjacent to the intestinal tract. The pencil beam algorithm was used for dose calculation.

### 4.4. Follow-Up Evaluation and Statistical Analysis

After IGPT, patients were followed up at 1.5, 3, 6, 9, and 12 months in the first year, at intervals of 3 months in the second year, and at intervals of 3–6 months in the third year and thereafter. Regular follow-up studies included physical examinations, a liver function test, tumor marker examinations, and dynamic abdominal CT or gadoxetate sodium-MRI. Lung CT was generally performed once a year, or whenever necessary in the case of lung metastasis. Definition of local failure was based on the modified Response Evaluation Criteria in Solid Tumors (> 20% increases in sum of diameters of contrast-enhanced parts).

OS, LC, and PFS rates were calculated using the Kaplan–Meier method from the start of IGPT to the last follow-up or death. Hazard ratios and 95% confidence intervals (CI) for OS and LC were estimated using univariate and multivariate Cox’s proportional hazards models. Acute and late adverse events were evaluated with Common Terminology Criteria for Adverse Events version 4.0. QOL scores were assessed using EORTC QCLQ-C3 version 3.0, QLQ-HCC18, and SF-36 before and 6 and 12 months after IGPT and analyzed using the Mann–Whitney U test and a one-way analysis of variance among the follow-up periods. *p*-values of < 0.05 were considered to be significant. SPSS 24.0 J (SPSS Japan Inc., Tokyo, Japan) was used for all analyses.

## 5. Conclusions

IGPT is safe and effective for HCC in elderly patients. As a new treatment modality, IGPT may become one of the standard treatments for elderly HCC. Since there was no reduction in QOL during/after treatment, treatment may be possible without changing daily living for elderly patients. A larger-scale comparative study using HRQOL needs to be conducted to compare the results obtained with those of other modalities.

## Figures and Tables

**Table 1:** Patient and tumor characteristics.

Variable	Level	Total [%]
Number	*N*	71
Age	Median (range)	82 (80–96)
Sex	Male/Female	43 (61)/28 (39)
Performance status	0/1/2/3	44 (62)/20 (28)/4 (6)/3 (4)
Major underlying liver disease	HBV/HCV/Alcoholic/NAFLD	7 (10)/28 (39)/5 (7)/31 (44)
Treatment options	Operable/RFA indicated/TACE indicated	27 (38)/12 (17)/46 (65)
Treatment history	−(primary tumor)/+(recurrence or IM)	46 (65)/25 (35)
Longest tumor diameter (mm)	Median (range)	32 (8–111)
Tumor volume (cc)	Median (range)	25.6 (1.2–602.5)
Child–Pugh classification	A5/A6/B7-9	49 (69)/15 (21)/7 (10)
TNM (UICC 8th)	T1N0M0/T2N0M0/T3bN0M0	58 (82)/11 (15)/2 (3)
BCLC staging system	0/A/B/C/D	7 (10)/45 (63)/1 (2)/15 (21)/3 (4)
Okuda staging system	I/II	62 (87)/9 (13)
Liver damage ^1^	A/B/C	47 (66)/22 (31)/2 (3)
^99m^Tc-GSA hepatic scintigraphy	HH15/LHL15 Median (range)	0.60 (0.48––0.85)/0.88 (0.69–0.94)
ICGR_15_ (%)	Median (range)	18.3 (5.0–47.4)
K-ICG (%)	Median (range)	0.12 (0.01–0.20)
ALBI grade	1/2–3	35 (49)/36 (51)
FIB-4 index	Median (range)	4.3 (1.6–15.2)
Serum hyaluronate level (ng/mL)	Median (range)	101 (5–1920)
Serum type IV collagen (ng/mL)	Median (range)	5.5 (2.9–495.0)
Alpha fetoprotein (ng/mL)	Median (range)	6.8 (1.6–55149.6)
Alpha fetoprotein-L3 (%)	Median (range)	1.4 (<0.5–96.3)
PIVKA-II (mAU/mL)	Median (range)	57 (8–91900)

^1^ Classification based on ascites, serum bilirubin, serum albumin, indocyanine green retention rate at 15 min (ICG-R15) (%), and prothrombin time activity (%) by the Liver Cancer Study Group of Japan; HBV = hepatitis B virus; HCV = hepatitis C virus; IM = intrahepatic metastasis; BCLC, Barcelona Clinic Liver Cancer; ^99m^Tc-GSA = technetium-99m diethylenetriamine-penta-acetic acid-galactosyl human serum albumin; HH15 = clearance index, which is the ratio of radioactivity of the liver at 15 min over that at 3 min after injection; LHL15 = the receptor index, which is the ratio of radioactivity of the liver over that of the liver plus heart at 15 min; K-ICG = plasma clearance rate of indocyanine green; UICC = Union for International Cancer Control; ALBI = albumin-bilirubin; FIB-4 = fibrosis-4; PIVKA-II = protein induced by vitamin K absence or antagonist-II.

**Table 2:** Treatment characteristics and dose volume analyses.

Variable	Level	Total [%]
Dose fractionation (peripheral/central)	66 GyRBE/10 Fr/72.6 GyRBE/22 Fr	47 (66)/24 (34)
Number of beam portals	2/3/4	8 (11)/61 (86)/2 (3)
Vicinity to the gastrointestinal tract	≤1 cm	25 (31)
PTV D_98_ (%)	Median (interquartile range)	90.5 (65.0–97.4)
PTV D_95_ (%)	Median (interquartile range)	95.9 (74.1–98.5)
Homogeneity index	Median (interquartile range)	0.11 (0.05–0.36)
Conformity index	Median (interquartile range)	1.26 (0.97–1.42)
Mean liver dose (Liver-GTV) (GyRBE)	Median (range)	12.3 (2.0–21.6)
Liver-GTV V25/V30 (peripheral) ^1^ (%)	Median	15.6/14.0
Liver-GTV V32/V38 (central) ^2^ (%)	Median	21.6/19.4
V < 1 GyRBE remnant liver volume	Standard liver volume ≥ 35%	71 (100)/0 (0)
Skin V80%/V50% (cc)	Median (range)	0 (0–22.8)/2.9 (0–53.7)
Colon D1cc/D10cc (GyRBE)	Median (range)	1.0 (0–53.5)/0 (0–47.9)
Stomach D1cc/D10cc (GyRBE)	Median (range)	0 (0–50.8)/0 (0–40.9)
Duodenum D1cc/D10cc (GyRBE)	Median (range)	0 (0–52.6)/0 (0–27.3)

^1^ Volume irradiated with dose constraints of Liver-GTV for the peripheral lesion (Table S1); ^2^ volume irradiated with dose constraints of Liver-GTV for the central lesion (Supplemental Table S1); GyRBE = gray relative biological effectiveness; Fr = fraction; PTV Dx = dose (%) to X% of the planning target volume; homogeneity index = (D_2_−D_98_)/D_50_, according to ICRU 83; Conformity index = the volume receiving at least 95% of the prescribed dose (V_95_)/the volume of the planning target volume (V_PTV_), according to ICRU 83; GTV = gross tumor volume; Vx = volume receiving at least X GyRBE or X% of the prescribed dose; Dxcc = dose delivered to X cc of the target volume.

**Table 3:** Uni- and multivariate Cox’s analyses for overall survival.

Factor	*N* of Data	*N* of event (%)	Level	Univariate HR (95% CI)	*p*	Multivariate HR (95% CI)	*p*
Age	71	28 (39)	≥ vs. <82 years old ^1^	0.75 (0.29, 1.97)	0.57		
Sex	71	28 (39)	Male vs. Female	2.86 (1.01, 8.13)	0.048	4.38 (1.02, 18.9)	0.047
Performance status	71	28 (39)	≥1 vs. 0	5.10 (1.81, 14.4)	0.002	7.66 (1.57, 37.3)	0.012
Major underlying liver disease	71	28 (39)	NAFLD vs. Others	0.74 (0.28, 1.96)	0.55		
Treatment history	71	28 (39)	+(recurrence or IM) vs.−(primary tumor)	2.91 (1.06, 7.98)	0.038	5.44 (0.95, 31.1)	0.057
Longest tumor diameter	71	28 (39)	≥ vs. <32 mm ^1^	0.87 (0.34, 2.23)	0.77		
Tumor volume	71	28 (39)	≥ vs. <25.6 cc ^1^	1.05 (0.40, 2.71)	0.92		
Child–Pugh score	71	28 (39)	A6, B7 vs. A5	3.27 (1.15, 9.31)	0.026	2.02 (0.48, 8.42)	0.34
TNM (UICC8th)	71	28 (39)	≥T2 vs. T1	1.40 (0.42, 4.72)	0.58		
Okuda staging	71	28 (39)	II vs. I	2.12 (0.52, 8.70)	0.30		
BCLC staging	71	28 (39)	B–D vs. 0, A	1.56 (0.54, 4.53)	0.41		
Liver damage	71	28 (39)	B vs. A	2.52 (0.92, 6.93)	0.073		
HH15	71	28 (39)	>0.55 vs. ≤0.55 ^2^	1.26 (0.41, 3.91)	0.69		
LHL15	71	28 (39)	<0.92 vs. ≥0.92 ^2^	1.62 (0.38, 6.88)	0.51		
ICGR_15_	71	28 (39)	≥10 vs. <10 % ^2^	1.71 (0.31, 9.50)	0.54		
K-ICG	71	28 (39)	<0.15 vs. ≥0.15, ≤0.22 %	1.62 (0.38, 6.88)	0.58		
ALBI grade	71	28 (39)	2, 3 vs. 1	1.53 (0.59, 4.00)	0.38		
FIB-4 index	71	28 (39)	≥2.67 vs. <2.67	0.86 (0.18, 4.15)	0.85		
Treatment options	71	28 (39)	Inoperable vs. operable	6.90 (2.05, 23.3)	0.002	2.03 (0.43, 9.59)	0.37
	71	28 (39)	RFA untreatable vs. treatable	0.89 (0.25, 3.16)	0.86		
	71	28 (39)	TACE untreatable vs. treatable	2.24 (0.83, 6.07)	0.11		
Dose fractionation	71	28 (39)	72.6 GyRBE/22 Fr vs. 66 GyRBE/10 Fr	1.49 (0.55, 4.06)	0.43		
Vicinity to the gastrointestinal tract	71	28 (39)	≤ vs. >1 cm	1.28 (0.47, 3.52)	0.63		
Alpha fetoprotein	71	28 (39)	>20 vs. ≤20 ng/mL ^2^	3.36 (1.22, 9.22)	0.019	2.05 (0.43, 9.73)	0.37
PIVKA-II	71	28 (39)	>40 vs. ≤40 mAU/mL ^2^	2.88 (1.04, 7.94)	0.042	3.55 (0.81, 15.5)	0.092
Local control	71	28 (39)	Failure vs. control	3.64 (0.83. 16.0)	0.09		

^1^ Cut-off value was the median value; ^2^ cut-off value was the upper limit of normal reference levels; HR = hazard ratio.

**Table 4:** Uni- and multivariate Cox’s analyses for local control.

Factor	*N* of Data	*N* of Event (%)	Level	Univariate HR (95% CI)	*P*	Multivariate HR (95% CI)	*P*
Age	71	9 (13)	≥ vs. <82 years old ^1^	2.88 (0.55, 15.0)	0.21		
Sex	71	9 (13)	Male vs. Female	0.74 (0.17, 3.24)	0.69		
Performance status	71	9 (13)	≥1 vs. 0	0.26 (0.06, 1.13)	0.07		
Major underlying liver disease	71	9 (13)	NAFLD vs. Others	0.96 (0.24, 3.94)	0.96		
Treatment history	71	9 (13)	+(recurrence or IM) vs.-(primary tumor)	1.04 (0.25, 4.24)	0.96		
Longest tumor diameter	71	9 (13)	≥ vs. <32 mm^1^	9.10 (1.07, 77.2)	0.043	1.10 (0.02, 73.5)	0.96
Tumor volume	71	9 (13)	≥ vs. <25.6 cc ^1^	10.4 (1.22, 88.0)	0.032	0.11 (0.002, 6.94)	0.30
Child–Pugh score	71	9 (13)	A6, B7 vs. A5	0.24 (0.03, 2.08)	0.20		
TNM (UICC8th)	71	9 (13)	≥T2 vs. T1	0.52 (0.06, 4.57)	0.57		
Okuda staging	71	9 (13)	II vs. I	0.84 (0.09, 7.67)	0.88		
BCLC staging	71	9 (13)	B–D vs. 0, A	2.51 (0.60, 10.6)	0.21		
Liver damage	71	9 (13)	B vs. A	0.21 (0.03, 1.81)	0.16		
HH15	71	9 (13)	>0.55 vs. ≤0.55 ^2^	0.33 (0.08, 1.41)	0.14		
LHL15	71	9 (13)	<0.92 vs. ≥0.92 ^2^	1.36 (0.15, 12.2)	0.78		
ICGR_15_	71	9 (13)	≥10 vs. <10 %^2^	0.86 (0.09, 8.08)	0.89		
K-ICG	71	9 (13)	<0.15 vs. ≥0.15, ≤0.22%	1.36 (0.15, 12.2)	0.78		
ALBI grade	71	9 (13)	2, 3 vs. 1	0.44 (0.10, 1.92)	0.27		
FIB-4 index	71	9 (13)	≥2.67 vs. <2.67	0.86 (0.09, 8.08)	0.89		
Treatment options	71	9 (13)	Inoperable vs. operable	2.37 (0.45, 12.3)	0.31		
	71	9 (13)	RFA untreatable vs. treatable	- ^3^	- ^3^		
	71	9 (13)	TACE untreatable vs. treatable	1.56 (0.38, 6.44)	0.54		
Dose fractionation	71	9 (13)	72.6 GyRBE/22 Fr vs. 66 GyRBE/10 Fr	2.83 (0.68, 11.72)	0.15		
Vicinity to the gastrointestinal tract	71	9 (13)	≤ vs. >1 cm	5.29 (1.19, 23.6)	0.029	0.25 (0.05, 1.22)	0.08
Alpha fetoprotein	71	9 (13)	> 20 vs. ≤20 ng/mL ^2^	2.34 (0.59, 10.1)	0.22		
PIVKA-II	71	9 (13)	> 40 vs. ≤40 mAU/mL ^2^	3.08 (0.59, 16.0)	0.18		

^1^ Cut-off value was the median value; ^2^ cut-off value was the upper limit of normal reference levels; ^3^ no patients with RFA treatable had local control failure.

**Table 5:** Acute and late complications related to image-guided proton therapy.

Adverse Event	BCLC 0–A 52 Patients (Grade 2/3)	BCLC B–D 19 Patients (Grade 2/3)	Total Number (Grade 2/3)
Acute toxicity			
Hepatobiliary enzyme	0/0	1/0	1/0
Cytopenia	0/0	0/0	0/0
Dermatitis	2/0	2/0	4/0
Decrease in Child–Pugh score ≥ 2	0	0	0
Radiation pneumonitis^1^	0/0	0/0	0/0
Late toxicity			
Hepatobiliary enzyme	0/0	0/0	0/0
Cytopenia	0/0	0/0	0/0
Dermatitis	0/0	0/1	0/1
Decrease in Child–Pugh score ≥ 2	1 ^2^	3 ^2^	4 ^2^
Radiation pneumonitis ^1^	0/0	0/0	0/0
Soft-tissue inflammation	1/0	0/0	1/0
Rib fracture	1/0	0/0	1/0

^1^ Pneumonitis occurring within the irradiation field within and after 3 months was defined as acute and late radiation pneumonitis, respectively; ^2^ potentially due to original cirrhosis and infection.

**Table 6:** Temporal changes in the quality of life score after image-guided proton therapy.

Variables	Pre-IGPT (*N* = 71)	6 Months (*N* = 58)	*p* ^1^	12 Months (*N* = 46)	*p* ^1^	*p* ^2^
EORTC QLQ-C30						
Global health status/QOL	51.4 (20.5)	52.0 (22.0)	0.91, 0.71	55.4 (22.2)	0.41, 0.12	0.69, 0.19
Functional scales						
Physical functioning	65.4 (24.6)	66.1 (26.7)	0.75, 0.78	62.6 (28.7)	0.81, 0.37	0.86, 0.66
Role functioning	72.3 (31.9)	66.5 (32.9)	0.18, 0.49	62.8 (34.5)	0.15, 0.78	0.25, 0.53
Emotional functioning	70.4 (23.5)	75.0 (21.8)	0.21, 0.53	75.2 (20.1)	0.33, 0.26	0.40, 0.37
Cognitive functioning	68.8 (23.0)	72.8 (20.3)	0.44, 0.22	65.5 (25.8)	0.59, 0.35	0.48, 0.54
Social functioning	76.5 (25.9)	77.5 (25.0)	0.89, 0.99	76.7 (28.7)	0.69, 0.08	0.93, 0.21
Symptom scales/items						
Fatigue	44.4 (25.1)	45.0 (24.3)	0.77, 0.53	43.7 (25.0)	0.93, 0.22	0.96, 0.28
Nausea and vomiting	3.3 (9.2)	4.1 (9.6)	0.46, 0.78	5.8 (10.2)	0.08, 0.95	0.21, 0.96
Pain	26.1 (27.7)	34.5 (33.5)	0.20, 0.83	30.2 (29.4)	0.45, 0.45	0.42, 0.75
Dyspnea	29.1 (26.4)	33.3 (25.2)	0.26, 0.23	33.3 (26.2)	0.31, 0.99	0.44, 0.38
Insomnia	27.2 (27.2)	28.1 (30.7)	0.93, 0.90	30.2 (33.2)	0.89, 0.99	0.98, 0.95
Appetite loss	23.0 (23.6)	25.1 (26.9)	0.78, 0.33	24.0 (29.4)	0.79, 0.43	0.90, 0.64
Constipation	29.6 (34.1)	28.7 (29.8)	0.86, 0.95	26.4 (27.8)	0.90, 0.76	0.96, 0.97
Diarrhea	13.1 (22.2)	12.3 (24.1)	0.55, 0.88	8.5 (14.7)	0.38, 0.26	0.65, 0.30
Financial difficulties	19.2 (26.2)	13.5 (21.7)	0.20, 0.24	17.1 (25.6)	0.62, 0.20	0.44, 0.19
EORTC QLQ-HCC18						
Symptom scale/items						
Fatigue	34.1 (23.7)	40.7 (28.3)	0.24, 0.59	41.3 (27.4)	0.21, 0.52	0.35, 0.85
Body image	31.2 (23.2)	34.5 (26.5)	0.55, 0.99	32.6 (20.2)	0.64, 0.76	0.81, 0.78
Jaundice	15.0 (17.6)	18.4 (18.0)	0.25, 0.38	15.9 (17.4)	0.83, 0.95	0.51, 0.80
Nutrition	17.7 (16.1)	18.8 (14.3)	0.48, 0.95	19.4 (13.7)	0.36, 0.68	0.61, 0.67
Pain	14.6 (17.6)	20.2 (22.2)	0.14, 0.53	19.4 (19.9)	0.14, 0.85	0.22, 0.51
Fever	5.4 (11.5)	10.8 (18.0)	0.04, 0.27	10.5 (19.9)	0.16, 0.97	0.10, 0.65
Single items						
Abdominal swelling	23.9 (28.8)	25.7 (32.1)	0.94, 0.57	24.0 (24.5)	0.66, 0.43	0.91, 0.55
Sex life	84.0 (33.7)	87.1 (30.0)	0.74, 0.73	84.5 (35.1)	0.81, 0.61	0.94, 0.74
SF-36						
Physical functioning	60.0 (26.3)	59.6 (27.7)	0.98, 0.88	55.9 (29.5)	0.55, 0.63	0.80, 0.75
Role physical	60.7 (31.4)	60.3 (33.2)	0.94, 0.85	53.5 (35.8)	0.33, 0.30	0.59, 0.57
Bodily pain	64.4 (25.9)	65.1 (29.8)	0.70, 0.75	64.4 (32.0)	0.91, 0.58	0.94, 0.85
General health	46.4 (19.2)	51.4 (20.4)	0.22, 0.98	53.9 (21.2)	0.07, 0.63	0.17, 0.87
Vitality	50.3 (24.6)	53.3 (22.8)	0.52, 0.80	50.9 (26.3)	0.82, 0.51	0.82, 0.55
Social functioning	69.5 (29.1)	70.8 (28.9)	0.77, 0.78	68.3 (30.4)	0.84, 0.76	0.91, 0.88
Role emotional	62.3 (34.4)	66.1 (33.1)	0.55, 0.90	57.4 (37.3)	0.57, 0.38	0.53, 0.64
Mental health	60.1 (24.1)	64.0 (21.0)	0.46, 0.80	63.8 (20.9)	0.52, 0.71	0.71, 0.96
3-Physical CS ^3^	37.8 (12.6)	37.5 (12.9)	0.81, 0.93	36.8 (13.4)	0.82, 0.71	0.96, 0.89
3-Mental CS ^3^	51.7 (9.6)	54.0 (9.4)	0.20, 0.93	55.7 (8.6)	0.03, 0.96	0.10, 0.94
3-Role-social CS ^3^	42.0 (13.6)	42.1 (14.7)	0.98, 0.88	37.9 (15.1)	0.14, 0.27	0.28, 0.49
2-Physical CS ^3^	34.9 (15.1)	34.6 (16.1)	0.93, 0.95	31.0 (16.5)	0.24, 0.34	0.46, 0.56
2-Mental CS ^3^	50.2 (10.0)	52.4 (9.5)	0.21, 0.75	53.3 (8.8)	0.13, 0.84	0.24, 0.98

^1^ Differences were examined by the Mann–Whitney U test between pre-PT and 6 or 12 months after IGPT for all patients (1st *p*) and BCLC B-D patients (2nd *p*); ^2^ Differences were examined by a one-way analysis of variance from pre-PT to 12 months after IGPT for all patients (1st *p*) and BCLC B-D patients (2nd *p*); ^3^ Scored using factor coefficients based on the 1995 Japan National Survey; CS = component summary; Data are mean (standard deviation).

## Data Availability

The data presented in this study are available on request from the corresponding author. The data are not publicly available due to institutional guidelines.

## Supplementary Materials

The following are available online at https://www.mdpi.com/2072-6694/13/2/219/s1, File S1: Details of treatment planning, Table S1: Dose constraints of the organ at risk.
