# Supplementary material for: Image-Guided Proton Therapy for Elderly Patients with Hepatocellular Carcinoma: High Local Control and Quality of Life Preservation
Source: Cancers (Basel). 2021 Jan 9;13(2):219. doi: 10.3390/cancers13020219 (PMC7827493; doi:10.3390/cancers13020219)
Supplement: Supplementary file 1 [file cancers-13-00219-s001.pdf]

cancers

MDPI

Supplementary materials: Image-Guided Proton Therapy for Elderly Patients with Hepatocellular Carcinoma: High Local Control and Quality of Life Preservation

Hiromitsu Iwata, Hiroyuki Ogino, Yukiko Hattori, Koichiro Nakajima, Kento Nomura, Kensuke Hayashi, Toshiyuki Toshito, Shigeru Sasaki, Shingo Hashimoto, Jun-etsu Mizoe and Yuta Shibamoto

File S1. Details of Treatment Planning

Patients were immobilized in the supine position with an ESFORM immobilization system (Engineering System, Nagano, Japan) and 2-mm-thick CT images were taken using a 16-row multi-detector CT (Aquilion LB; Toshiba Medical Systems, Tochigi, Japan) during the expiration phase for treatment planning. Additional scans were performed during the expiration phase after administering iopamidol or iohexol (370 or 300 mgI/L) through a forearm vein using a power injector at a rate of 3 mL/second. Scans were performed 30 and 100 seconds after the injection. All patients then underwent CT simulation with 4DCT to account for tumor motion with deformation. Patient respiratory waveforms were monitored throughout the procedures and recorded using an AZ-733 V respiratory gating system (Anzai Medical, Tokyo, Japan). After CT simulation, 4DCT images were reconstructed into 10 respiratory phases, with the end of expiration defined as phase 50% and the end of inspiration as phase 0% (=100%).

MRI was performed on a 1.5-T system with an 8-channel phased-array coil using gadolinium ethoxybenzyl diethylenetriaminepentaacetic acid (EOB). After several sequences were obtained, dynamic and hepatocyte phase images were obtained using fat-suppressed T1-weighted gradient-echo images with a 3D acquisition sequence named VIBE (volumetric interpolated breath-hold examination) before and after the intravenous bolus administration of EOB (0.1 mL/kg of body weight) at a rate of 1.0 mL/second, followed immediately by saline flushing. Hepatobiliary phase imaging was obtained 15 minutes after the start of the injection.

MRI performed during the arterial and hepatobiliary phases was fused with CT images using MIM Maestro (version 6.9.6, MIM Software Inc., Cleveland, OH, USA). Visible lesions on CT and/or MRI were taken as the gross tumor volume (GTV), and the clinical target volume (CTV) was equal to GTV. In addition to CTV, CTVs from 4DCT were contoured on CT images of each respiratory phase (CTV0, CTV10 to CTV90). The internal clinical target volume (ICTV) was defined as the envelope of CTV. ICTV was divided into ICTV-all, consisting of CTVs across all respiratory phases, and ICTV-gate, used only for patients whose tumor moved more than 10 mm in any direction, comprising CTV within the gating window around phase 50%. The gating window was selected with reference to the amplitude of the marker and tumor movement. ICTV for each beam was expanded laterally from the perspective of the beam to encompass the set-up error margin (SM) and internal motion margin (IM). The lateral margin was 6 mm, which included SM and only intrafractional IM. In addition, beam-specific distal and proximal margins to ICTV were assigned along the beam path axis to account for uncertainties in the range of proton beams. Margins were typically 4–8 mm, based on the definition of Moyers et al. [43]. Compensation boluses were used to adjust the proton beam range and bolus smearing margins were typically 10–14 mm [43]. Lateral and beam-specific margins were slightly adjusted to meet the dose constraints.

Table S1. Dose constraints of the organ at risk.

Organ at Risk	Dose Limit (GYRBE) (66 GYRBE/10 FR)	Dose Limit (GyRBE) (72.6 GyRBE/22 Fr)	Volume	EQD2 (GyRBE)
Liver-GTV	25.5	35	Dmean	28–32
	30 [1], 25 [2]	38 [1], 32 [2]	< 30%	36, 28
	Liver-GTV volume receiving ≤ 1 GyRBE exceeds 35% of the standard liver volume [3]			
Cord	36	48	Max	47–50
Duodenum	42	55	≤ 1 cc	60.5
	38	50	≤ 10 cc	52
Small intestine	42	55	≤ 1 cc	60.5
	38	50	≤ 10 cc	52
Stomach	42	55	≤ 1 cc	60.5
	38	50	≤ 10 cc	52
Colon	44	57	≤ 1 cc	64
	40	52.5	≤ 10 cc	56
Heart	58	64	≤ 10 cc	76
	24	30	≤ 10%	26
Kidney	20	25	≤ 33.3%	20
Esophagus	49	65	≤ 1 cc	78

[1] normal liver function; [2] cirrhosis; [3] 706.2 × body surface area (m^2) + 2.4 (mL); dose constraints were selected referencing the QUANTEC summary [45] and tolerable doses adopted at other domestic proton facilities; an α/β ratio of 3 GyRBE was used to calculate EQD2; the use of the linear-quadratic (LQ) formalism is not recommended in hypofractionated radiotherapy; however, the errors associated with the use of the LQ model may be small when the fraction number is ≥ 7 [47]; restrictions considering each internal motion were set as the planning organ at risk for all organs; GyRBE = gray relative biological effectiveness; Fr = fraction; EQD2 = equivalent total dose in the 2-GyRBE fraction; GTV = gross tumor volume; Dmean = mean dose.
